# Laparoscopic-based perivascular renal sympathetic nerve denervation: a feasibility study in a porcine model

**DOI:** 10.1186/s40001-020-00422-5

**Published:** 2020-06-18

**Authors:** Linwei Zhao, Enyong Su, Xiaohang Yang, Binbin Zhu, Zhiqiang Fan, Xianpei Wang, Datun Qi, Lijie Zhu, Mingfu Bai, You Zhang, Qiuping Zhao, Muwei Li, Chuanyu Gao

**Affiliations:** 1 Department of Cardiology, Zhengzhou University People’s Hospital, Zhengzhou, 450003 People’s Republic of China; 2 Department of Cardiology, Fuwai Central China Cardiovascular Hospital, Zhengzhou, 450003 People’s Republic of China; 3 Henan Provincial Key LAB for the Control of Coronary Heart Disease, Zhengzhou, 450003 People’s Republic of China; 4 Department of Urinary Surgery, Zhengzhou University People’s Hospital, Zhengzhou, 450003 People’s Republic of China; 5 Department of Hypertension, Fuwai Central China Cardiovascular Hospital, Zhengzhou, 450003 People’s Republic of China; 6 Henan Institute of Cardiovascular Epidemiology, Zhengzhou, 450003 People’s Republic of China; 7 Department of Cardiology, Zhengzhou 7th People’s Hospital, Zhengzhou, 450003 People’s Republic of China

**Keywords:** Laparoscope, Renal sympathetic nerve denervation, Hypertension

## Abstract

**Background:**

This study aims to evaluate the effects and safety of laparoscopic-based perivascular renal sympathetic nerve denervation (RDN) in a porcine model fed a high-fat diet.

**Method:**

Thirty-six high-fat diet-fed Bama minipigs were randomly divided into an RDN group (*n* = 18), in which minipigs received laparoscopic-based perivascular RDN, and a sham group (*n* = 18). All pigs were fed the high-fat diet after the operation to establish a model of obesity-induced hypertension. Bama pigs in the RDN and sham groups were killed at 3 time points [2 days after RDN (*n* = 6), day 90 (*n* = 6) and day 180 (*n* = 6)].

**Result:**

The systolic blood pressure (SBP) and noradrenaline (NE) concentration in the kidney tissue were significantly lower in the RDN group than in the sham group at 2 days (113.83 ± 3.26 mmHg vs 129.67 ± 3.32 mmHg, *P* = 0.011, and 112.02 ± 17.34 ng/g vs 268.48 ± 20.61 ng/g, *P* < 0.001, respectively), 90 days (116.83 ± 3.88 mmHg vs 145.00 ± 4.22 mmHg, *P* = 0.001, respectively) and 180 days (129.33 ± 2.87 mmHg vs 168.57 ± 2.86 mmHg, *P* < 0.001, and 152.15 ± 16.61 ng/g vs 318.97 ± 24.84 ng/g, *P* < 0.001, respectively) after the operation. The diastolic blood pressure (DBP) was significantly lower in the RDN group than in sham group at 90 and 180 days after the operation (72.17 ± 2.7 mmHg vs 81.50 ± 2.22 mmHg, *P* = 0.037, and 76.83 ± 2.75 mmHg vs 86.33 ± 2.22 mmHg *P* = 0.021, respectively). Based on the pathological evaluation, the renal sympathetic nerve fascicles were successfully disrupted by radiofrequency energy after laparoscopic-based perivascular RDN, but the intima was intact. Tyrosine hydroxylase (TH) expression was decreased, while the expression of the S100 protein was increased in treated renal arteries after RDN.

**Conclusions:**

Laparoscopic-based perivascular RDN prevented the occurrence and development of hypertension, and thus it may be an efficient and safe method for controlling blood pressure in an experimental model.

## Background

Renal sympathetic nerves are considered to play an important role in the progression of hypertension [1]. Methods that disrupt the renal sympathetic nerve have been considered a promising approach to treating hypertension by suppressing the overactivated sympathetic nervous system since the 1930s [2].

Currently, percutaneous endovascular catheter-based renal sympathetic nerve denervation (RDN) has been regarded as a promising method for the treatment of resistant hypertension [3]. With minimal surface wounding, this technique is proposed to destroy the renal sympathetic nerve contained in the arterial wall, suppress the overactivated sympathetic nervous system, lower blood pressure and decrease the intake of medicine by patients suffering from hypertension. However, the results of these studies remain controversial based on the results of the outcome of the Symplicity HTN-3 trial [4]; the positive findings of the SPYRAL HTN-ON MED [5] and RADIANCE-HTN SOLO [6] trials published in LANCET and presented at EURO PCR in May 2018 have provided some insights into the effectiveness of RDN. Comparing the results of different studies, the ablation devices used in these studies are presumed to be one of the significant factors contributing to the success of RDN. A reliable ablation method may be necessary for a successful RDN procedure. Since renal sympathetic nerves are mainly located near the adventitia and are not distributed through the intima of renal arteries based on anatomy studies, we therefore attempted to perform laparoscopic-based perivascular RDN in this study. The purpose of this study was to evaluate the safety and efficacy of laparoscopic-based perivascular RDN in a porcine model.

## Materials and methods

Thirty-six male Bama pigs (8-month old, 22.42 ± 0.78 kg) were purchased from Beijing Strong Century Minipigs Breeding Base [Beijing, China, approval number: SYXK(jing) 2018-0040]. All pigs were maintained at a controlled temperature and humidity on a 12:12-h dark–light cycle. All experimental procedures were approved by the Animal Care Ethics Committee of the Henan Provincial People’s Hospital, and were conducted in accordance with the American Physiology Society’s “Guides for the Care and Use of Laboratory Animals” published by the National Institutes of Health. Thirsty-six Bama swine were randomly assigned into two groups: an RDN group (*n* = 18) and a sham group (*n* = 18). All animals were fed separately. A high-fat (4100 kcal/kg) diet consisting of protein (10%), fat (41%), carbohydrates (43%) and minerals (6%) was initiated on the first day after RDN. The daily high-fat diet intake was approximately 5% of the body weight of each animal, and was adjusted dynamically every 2 weeks.

Before the operations, animals were provided a liquid diet for 2 days, followed by fasting for 12 h with free access to water. Venous blood was collected for measurements of renal function and lipid levels while pigs were under general anesthesia. Anticoagulant therapy was provided by an initial unfractionated heparin bolus (100 IU/kg IV), and 1000 IU was supplemented every additional hour. Renal arterial angiography and optical coherence tomography (OCT) (Light-Lab Imaging, Inc., USA) were performed to record the images of both renal arteries before the operation. Each minipig in the RDN group (*n* = 18) underwent bilateral laparoscopic-based perivascular RDN, and every minipig in the sham group (*n* = 18) underwent the same procedure except for radiofrequency ablation. Renal arterial angiography and OCT were performed again for follow-up before all swine were killed.

Follow-up renal arterial angiography and OCT were performed to identify injury to the endothelium, lumen stenosis, thrombosis and any other abnormalities in the renal arteries of 6 pigs in the sham group and 6 pigs in the treatment group prior to killing at 3 time points [2 days (*n* = 6), 90 days (*n* = 6) and 180 days (*n* = 6) after RDN]. Immediately thereafter, the pigs were heparinized and euthanized under deep anesthesia. The kidneys were removed and stored in liquid nitrogen, and the renal arteries were removed and stored in formalin and liquid nitrogen for further processing prior to pathological and molecular biological detection, respectively.

### Anesthesia and surgical technique

The pigs were sedated by administering an intramuscular injection of ketamine hydrochloride (5 mg/kg), midazolam (0.5 mg/kg), and Atropine (0.5 mg) to induce anesthesia, and an infusion channel was established by piercing the edge of the vein. Intravenous supplemental propofol (3 mg/kg), sufentanil (1 g/kg) and vecuronium bromide (0.1 mg/kg) were administered before endotracheal intubation and connected to the anesthesia machine. Sevoflurane (0.5–1.5 mac) was inhaled throughout the operation and its flow was controlled through the anesthesia ventilator (Dräger Fabius, Germany). The ventilator parameters were set as follows: tidal volume 12–15 ml/kg, respiratory rate 16–18 breaths/min, and a suction/inhalation ratio of 1:2 to maintain anesthesia. This technique was used for anesthesia and monitored by a senior anesthesiologist.

**Fig. 1:**
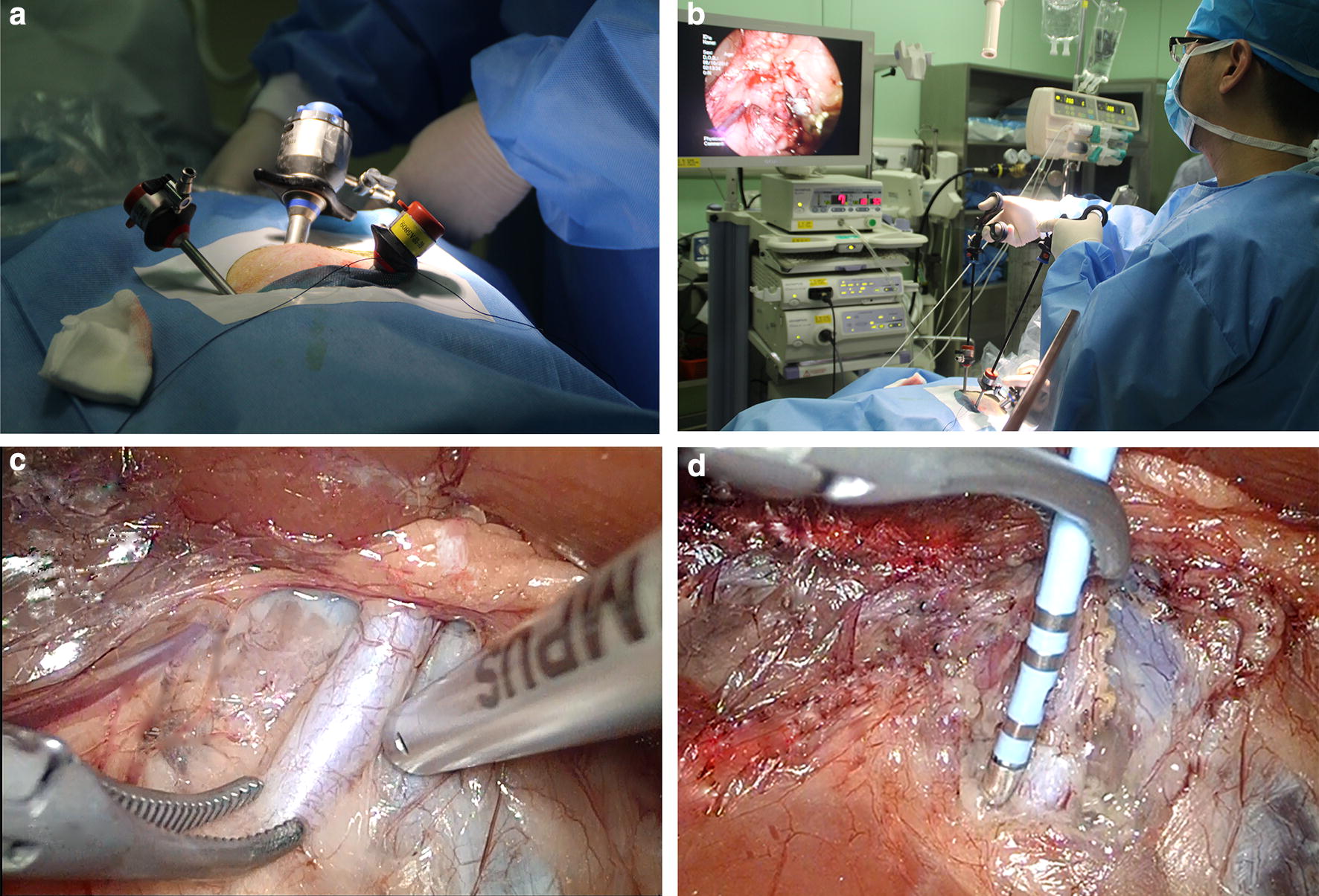
The procedures used during laparoscopic-based perivascular renal sympathetic nerve denervation (**a**–**d**)

Pigs were placed in the lateral position and secured with straps. The first incision was made approximately 2 cm below the intersection of the posterior axillary line and rib margin, and pneumoperitoneum was achieved with CO_2_ insufflation. The second incision was made approximately 2 cm above the intersection of the midaxillary line and spina iliaca. The third incision was made symmetric to the first incision in the anterior axillary line. A 10-mm trocar was then inserted in the second incision. Subsequently, two 5-mm trocars were inserted into the first and the third incisions (Fig. 1). A celioscope lens was introduced through the 10-mm trocar and 2 graspers were introduced through the two 5-mm trocars. After the selected renal artery was exposed and separated by the 2 graspers, a 7 Fr diameter radiofrequency (RF) ablation catheter (Biosense Webster, Diamond Bar, CA, USA), which was connected with an RF generator (Biosense Webster, Diamond Bar, CA, USA), was introduced to the retroperitoneum through a 5-mm trocar, and carried by a grasper. We applied the catheter for discrete radiofrequency ablations of 8 W or less lasting up to 120 s each to obtain up to six ablations separated both longitudinally and rotationally from the adventitia of the renal arteries. A saline solution was injected into the top of the RF catheter to control the temperature. After the ablation of one renal artery, the incisions were sutured layer by layer, and then the contralateral renal artery was ablated as well. After surgery, gentamicin sulfate (10 mg/kg) was administered intramuscularly for 7 days. This technique was performed by experienced urologists.

### Body weight and detection of blood biochemical parameters

Fasting pigs were weighed before the operation and every 2 weeks thereafter. Blood samples were collected from the superior vena cava after anesthesia at the 4 time points described above. Serum creatinine, total cholesterol (TC) and triglyceride (TG) levels were detected using an automatic biochemical analyzer (Rayto, Shenzhen, China). Serum neutrophil gelatinase-associated lipocalin (NGAL) and cystatin C levels and renal noradrenaline (NE) concentrations were detected using enzyme-linked immunosorbent assay (ELISA) kits (USCN Business Co. Ltd., Wuhan, China).

### Verification of laparoscopic-based perivascular renal nerve denervation

BP was measured using a BP-2010E monitor (Softron, China) at baseline and 2, 90 and 180 days after surgery to verify the effectiveness and safety of the laparoscopic-based perivascular RDN procedure, and the change in BP compared with the baseline (ΔBP) was also calculated. The NE concentration in the renal tissue was detected using an ELISA kit (USCN Business Co. Ltd., Wuhan, China).

Renal arteries stored in formalin were embedded in paraffin and sliced into 5-μm sections. Slides containing the renal vessels were stained with hematoxylin and eosin (HE) to evaluate the microscopic structures of the renal sympathetic nerve fascicles and the arterial wall. In addition, immunohistochemical staining for the S100 protein (ab868, Abcam, Cambridge, UK) and TH (tyrosine hydroxylase) (ab75875, Abcam, Cambridge, UK) was performed. An independent, experienced neuropathologist examined all histology sections for evidence of injury to the nerve, renal arterial walls and peri-arterial renal connective tissue containing the renal nerves in a blinded manner.

The renal arterial tissue was homogenized in RIPA buffer (Biotime, Beijing, China) using a Polytron homogenizer and incubated at 4 °C for 30 min. Lysates were centrifuged at 12,000*g* for 10 min at 4 °C; the supernatants were collected and the concentrations were determined using the BCA method. Equal amounts of solubilized proteins were separated on 10% polyacrylamide SDS gels and transferred onto polyvinylidene difluoride membranes (Millipore, USA). Membranes were incubated with 5% nonfat milk for 1 h at room temperature and then incubated with the indicated antibodies, rabbit anti-S100 (diluted 1:500), rabbit anti-TH (diluted 1:1000) (both from Abcam, USA) or rabbit anti-GAPDH (diluted 1:500) (Biotime, China), overnight at 4 °C. The membranes were incubated with a horseradish peroxidase-conjugated anti-rabbit secondary antibody (Biotime, China, diluted 1:2000 in TBST) for 1 h at room temperature and then detected using an ECL Kit (Millipore, USA) in 3 separate experiments, and the average of the 3 values was recorded.

### Statistical analysis

Body weights of pigs, BP, ΔBP, serum TC/TG, Cr, NGAL, cystatin C, S100 protein, and TH levels in the renal arterial wall and NE levels in the renal tissue were compared between the RDN group and sham group at the same time points. Continuous data are presented as means ± standard errors (SE). The normality of the distribution was assessed with the Shapiro–Wilk test. Variables with normal distributions were compared using *t* tests, whereas variables with skewed distributions were compared with the Mann–Whitney *U* test. All statistical analyses were performed with SPSS 20.0 software (SPSS Inc., Chicago, IL, USA). *P* values < 0.05 were considered statistically significant.

## Result

All 36 pigs underwent surgery. Of these animals, 18 underwent bilateral laparoscopic-based perivascular RDN, and the remaining pigs underwent sham operations. No side effects were observed and no pigs died unexpectedly during the experiment. Each artery in the RDN group was ablated at 6 points longitudinally and rotationally, the ablation points were uniformly distributed in the main renal artery, and the ablation time of every point was 120 s. The mean energy delivered to the tissues was 7.9 ± 0.43 W, the temperature was 43.95 ± 1.45 °C and the impedance was 210.78 ± 4.71 Ω.

### Body weight, serum total cholesterol, triglyceride, creatinine, cystatin C and neutrophil gelatinase-associated lipocalin levels

**Fig. 2:**
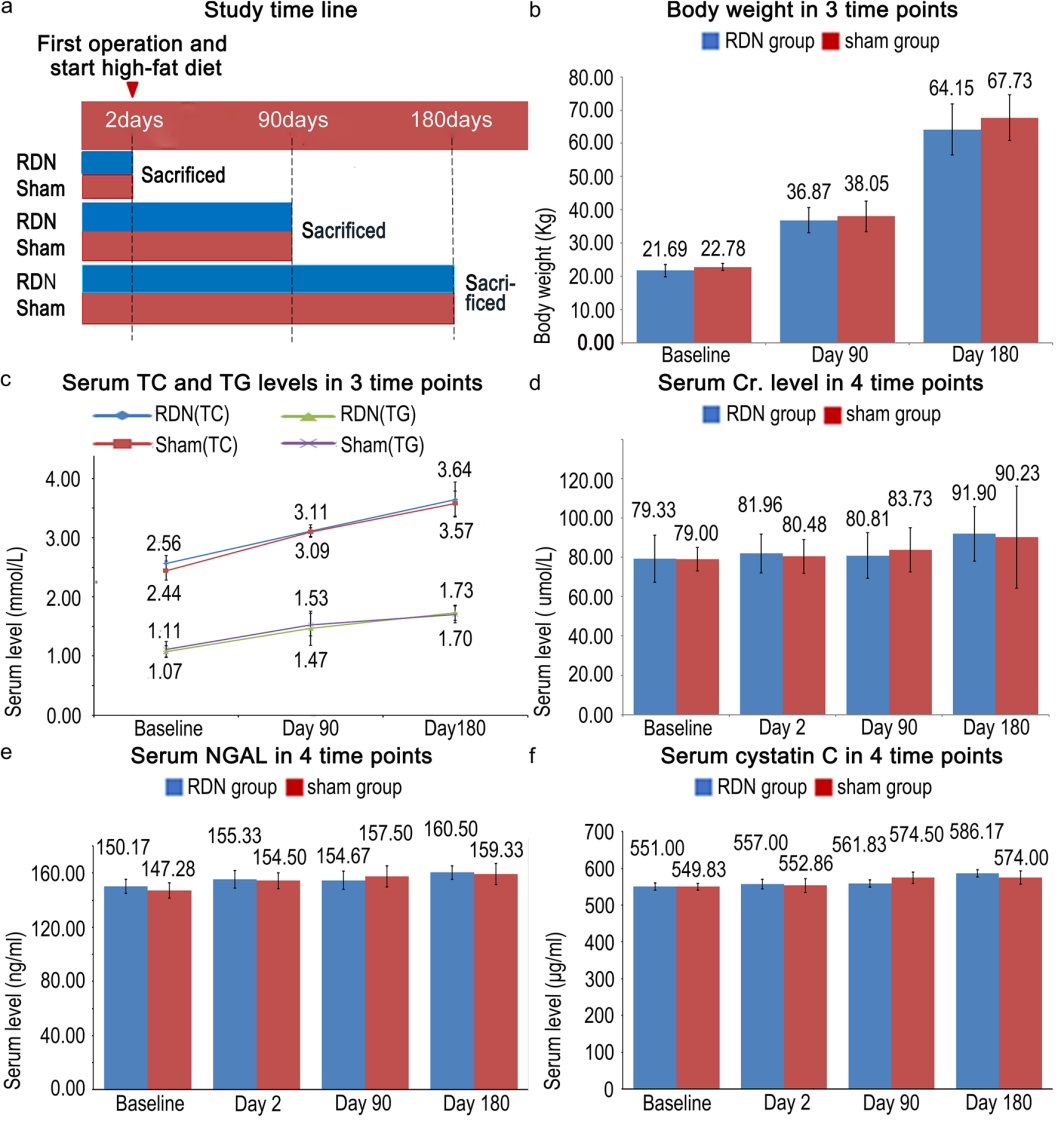
Study time line and changes in body weight, lipid levels and the Cr level before and after RDN. **a** Study time line. **b** Changes in body weight at 3 time points. **c** Changes in serum TC and TG levels at the 4 time points, and **d** changes in the serum Cr level at the 4 time points. **e** Changes in the serum NGAL in 4 time points. **f** Changes in the serum c in 4 cystatin C in 4 time points. *P < 0.05 compared with baseline values in the sham group

After the pigs consumed the high-fat diet, body weight and serum TC and TG levels were significantly increased. The body weight of the Bama pigs increased significantly from 21.69 ± 0.78 kg at baseline to 64.15 ± 3.12 kg (*P* < 0.001) at day 180 in the RDN group, but the difference was not significant compared with the sham group. Serum TC and TG levels were increased from 2.56 ± 0.14 mmol/l and 1.07 ± 0.10 mmol/l at baseline to 3.64 ± 0.29 mmol/l (*P* = 0.004) and 1.73 ± 0.13 mmol/l (*P* = 0.001), respectively, at day 180 in the RDN group, but were not significantly different from the values of the sham group. The serum creatinine, cystatin C and neutrophil gelatinase-associated lipocalin (NGAL) levels were not significantly different between the two groups (Fig. 2).

### Changes in blood pressure at the 4 time points

Before the surgery and high-fat diet feeding, the baseline systolic blood pressure was 127.67 ± 2.67 mmHg in the RDN group and 128.78 ± 2.08 mmHg in the sham group (*P* = 0.743), while diastolic blood pressure was 75.61 ± 1.70 mmHg in the RDN group and 74.50 ± 2.87 mmHg in the sham group, and the differences in SBP and DBP were not significant between the two groups (*P* = 0.678).

Two days after the surgery, SBP and ΔSBP were significantly lower in the RDN group than in sham group (113.83 ± 3.26 mmHg vs 129.67 ± 3.32 mmHg, *P* = 0.011, and − 15.00 ± 3.77 mmHg vs 3.33 ± 2.68 mmHg, *P* = 0.005, respectively), while non-significant differences in both DBP and ΔDBP (70.83 ± 2.54 mmHg vs 73.50 ± 2.95 mmHg, *P* = 0.254, and − 4.83 ± 2.22 mmHg vs − 0.50 ± 2.28 mmHg, *P* = 0.102, respectively) were observed.

At day 90, the values of SBP, ΔSBP, DBP and ΔDBP in the RDN group were significantly lower than in the sham group (116.83 ± 3.88 mmHg vs 145.00 ± 4.22 mmHg, *P* = 0.001, − 7.17 ± 4.28 mmHg vs 11.67 ± 3.61 mmHg, *P* = 0.012, 72.17 ± 2.7 mmHg vs 81.50 ± 2.22 mmHg, *P* = 0.037, and − 3.00 ± 3.33 vs 7.5 ± 3.08 mmHg, *P* = 0.022, respectively).

**Fig. 3:**
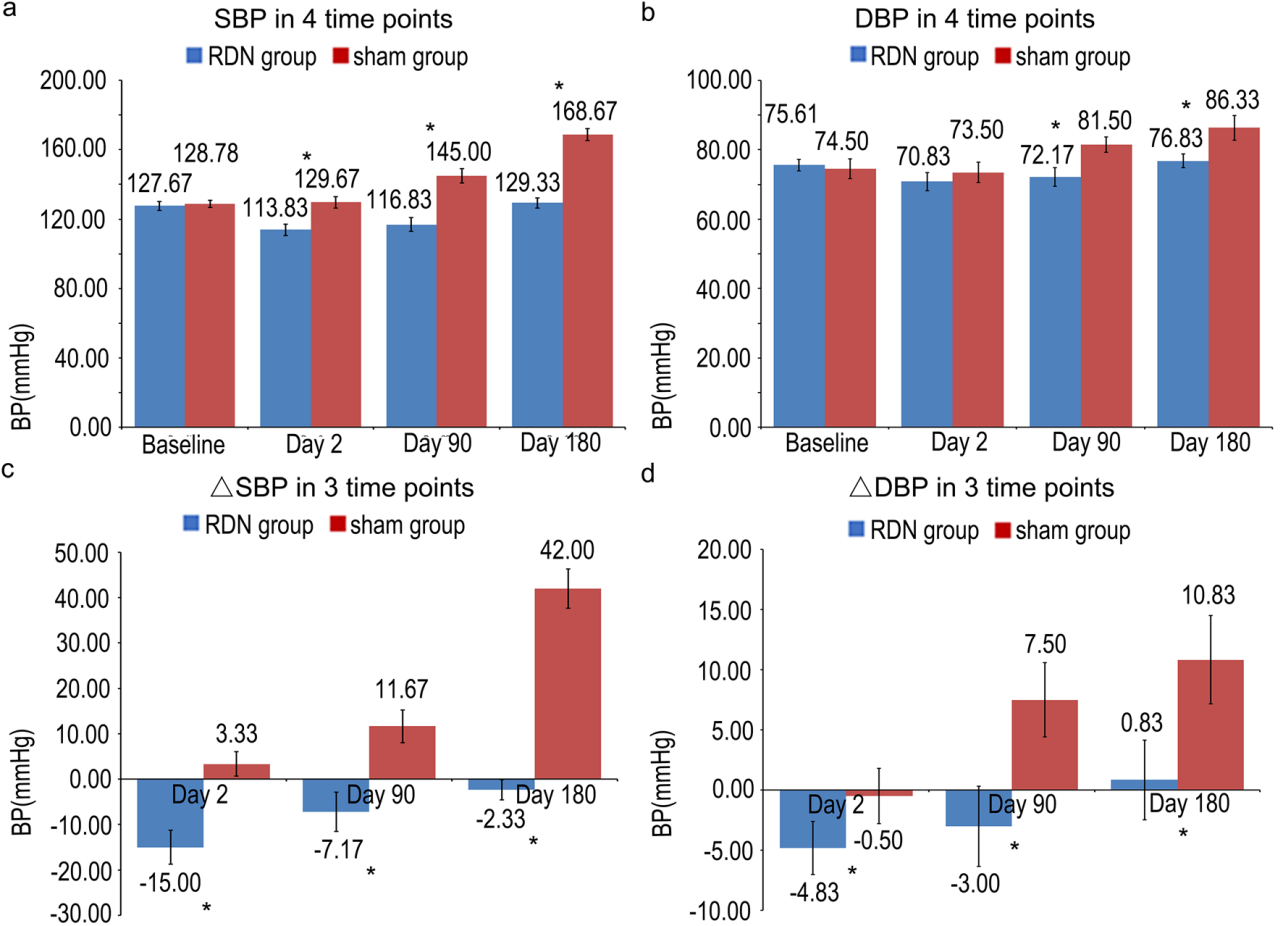
Changes in BP before and after RDN. **a** SBP at the 4 time points. **b** DBP at the 4 time points. **c** ΔSBP at 3 time points, and **d** ΔDBP at 3 time points. *P < 0.05 compared with baseline values in the sham group

At day 180, significantly lower SBP, ΔSBP, DBP and ΔDBP were observed in the RDN group than in the sham group (129.33 ± 2.87 mmHg vs 168.57 ± 2.86 mmHg, *P* < 0.001, − 2.33 ± 2.19 mmHg vs 42.00 ± 4.34 mmHg, *P* < 0.001, 76.83 ± 2.75 mmHg vs 86.33 ± 2.22 mmHg, *P* = 0.021, and 0.833 ± 3.33 mmHg vs 10.83 ± 3.66 mmHg, *P* = 0.035, respectively) (Fig. 3).

### Arteriography, optical coherence tomography and pathological evaluation of the atrial lumen and arterial wall

**Fig. 4:**
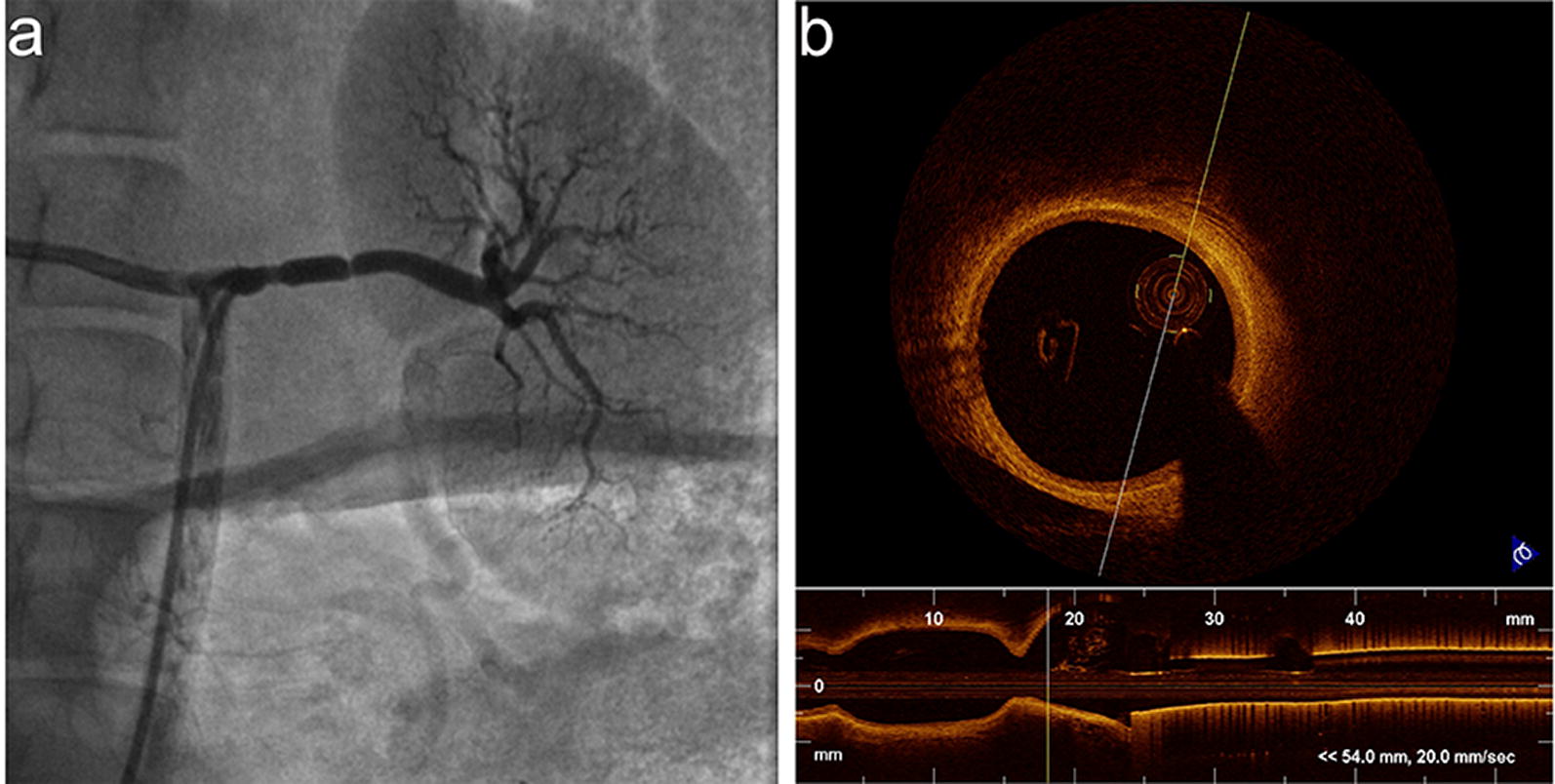
Representative images of renal arteries immediately after laparoscopic-based perivascular RDN. Spasms were observed using (**a**) RA and (**b**) OCT

**Fig. 5:**
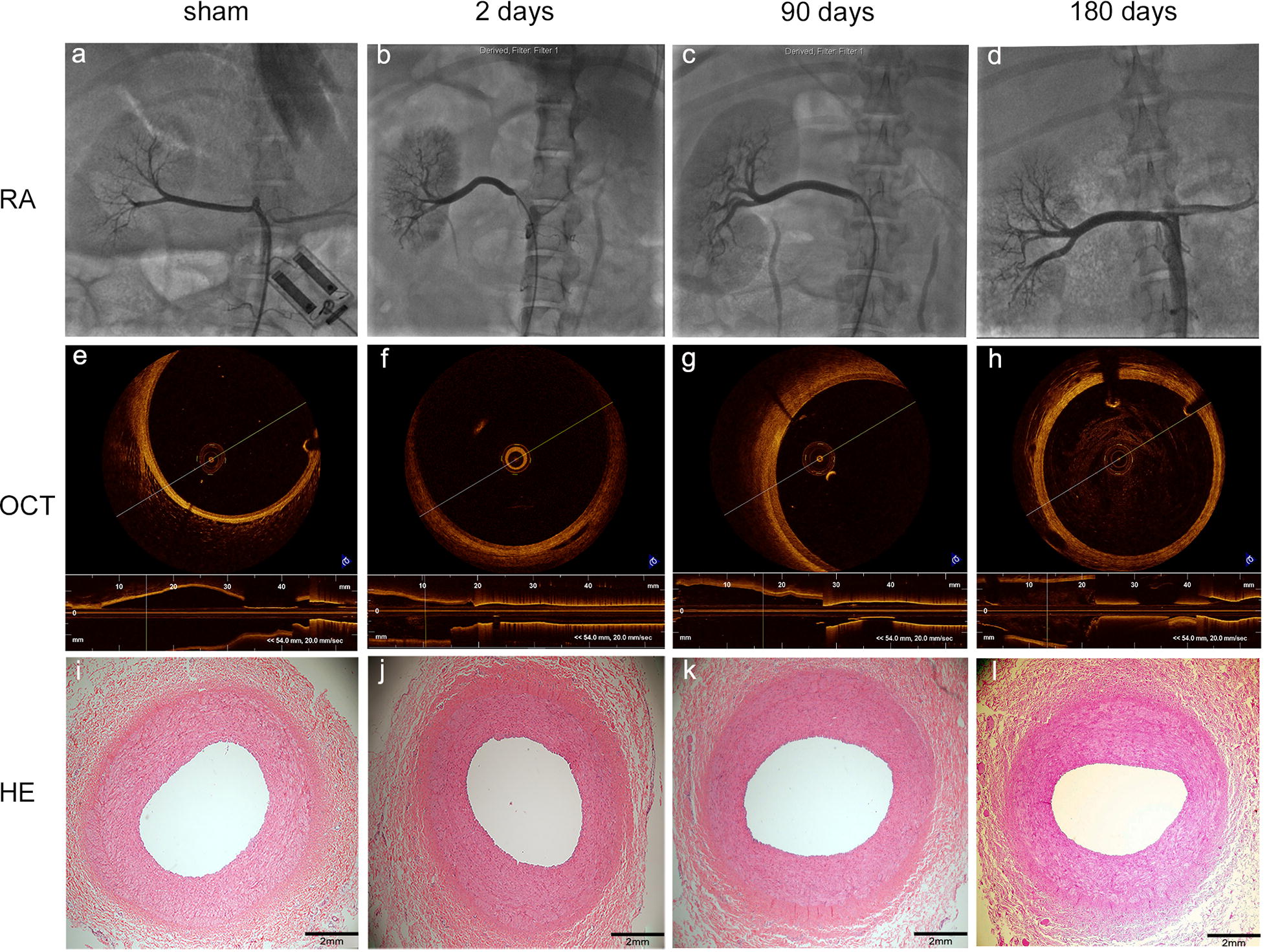
Representative images of renal arteries in the sham group and RDN group captured on days 2, 90 and 180 after surgery. **a**, **e**, **i** Representative images of untreated renal arteries (RA, OCT, and HE staining, respectively); the renal arterial wall appears intact without evidence of injury or inflammation. **b**, **f**, **j** Representative images of the renal artery 2 days after RDN (RA, OCT, and HE staining, respectively); no absolute injury was observed in the arterial wall using HE staining. **c**, **g**, **k** Representative images of the renal artery 90 days after RDN (RA, OCT, and HE staining, respectively); no spasms, stenosis, plaques or dissection were observed. **d**, **h**, **l** Representative images of the renal artery 180 days after RDN (RA, OCT, and HE staining, respectively); no spasms, stenosis, plaques or dissection were observed

Arteriography is the gold standard for identifying vessel narrowing of the vascular lumen, while OCT, which has a high axial resolution of 10–20 μm, accurately visualizes arterial wall lesions. All renal arteries were assessed using arteriography and OCT scanning. Spasms were immediately observed after laparoscopic-based perivascular RDN (Fig. 4), but no spasms were observed during subsequent assessments on days 2, 90 and 180. No aneurysmal changes, thrombi or other abnormalities were noted in the lumen or arterial wall in the 180-day study. HE staining did not reveal thrombi, dissections, aneurysms, perforations, hematomas, neointimal formation, or negative remodeling. Representative images of the arterial lumen at different time points are shown in Fig. 5.

### Pathological and immunohistochemical evaluations of nerve fascicles

Two days after laparoscopic-based perivascular RDN, HE staining of the affected arterial section indicated that nerve fascicles surrounding renal arteries exhibited vacuolization and nuclear pyknosis, and the endoneurium became disorganized. In contrast, the tissues of the sham group were intact.

The S100 protein is a marker of Schwann cells (SCs), which wrap around the axons of nerve cells. TH is the rate-limiting enzyme involved in catecholamine synthesis within the postganglionic nerve terminals, and TH expression has also been used as a functional marker of the activity of renal sympathetic nerve fascicles. Immunohistochemical staining for the S100 protein and TH protein revealed no significant differences between the sham group and RDN group 2 days after laparoscopic-based perivascular RDN.

At day 90, the affected nerve epineurium resembled a thick layer, the nerve bundles had atrophied, and endoneurial and perineural fibrosis were observed. Stained sections from the RDN group showed a marked decrease in levels of the TH protein but an increase in levels of the S100 protein compared with the sham group.

**Fig. 6:**
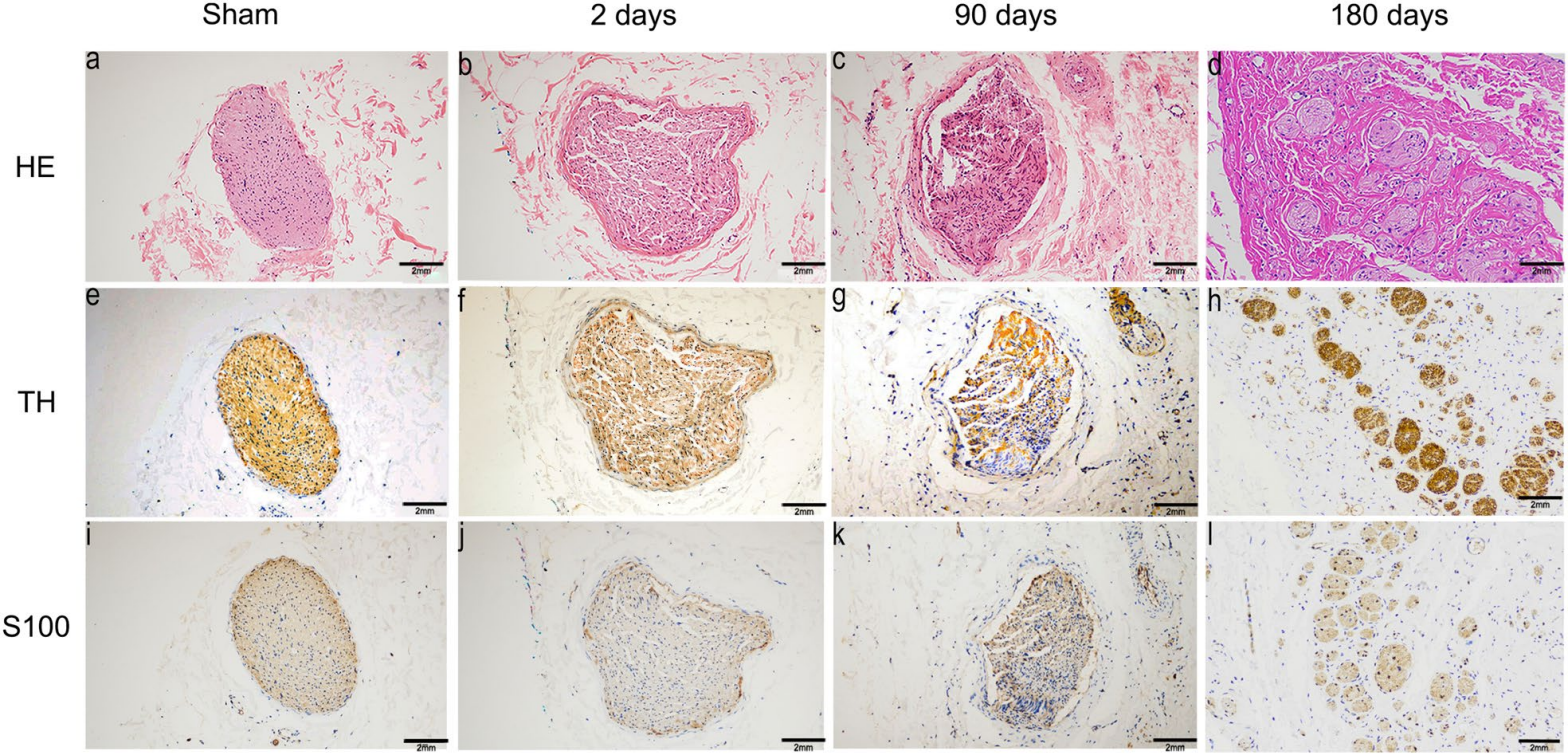
Representative images of pathological sections of renal nerves from the sham group and RDN group at 2, 90 and 180 days after surgery. **a**, **e**, **i** Representative images of untreated nerves (HE staining and immunohistochemical staining for TH and S100, respectively); normal nerve fascicles surrounded by a thin perineurium and epineurium were observed in the untreated renal artery, and immunohistochemical staining for TH and S100 protein showed moderate expression. **b**, **f**, **j** Representative images of nerve fibers at 2 days after RDN (HE staining and immunohistochemical staining for TH and S100, respectively). A broken perineurium surrounded the atrophic renal nerve and no inflammatory component was observed; immunohistochemical staining for TH and the S100 protein showed no obvious differences from the tissue before RDN. **c**, **g**, **k** Representative images of nerve fibers at 90 days after RDN (HE staining and immunohistochemical staining for TH and S100, respectively). The epineurium and endoneurium are thickened and fibrosis is observed in the perineural tissue and nerve fascicles; immunohistochemical staining showed a slight decrease in TH expression and an increase in S100 expression. **d**, **h**, **l** Representative images of nerve fascicles at 180 days after RDN (HE staining and immunohistochemical staining against TH and S100, respectively). Some newly regenerated nerve fascicles were observed. Immunohistochemical staining for TH and S100 showed moderate expression

At day 180, the thick layer, endoneurial and perineural fibrosis were also observed. In addition, some disordered nerve regrowth mixed with connective tissue was observed at the site of radiofrequency ablation, along with a decrease in immunohistochemical staining for the S100 protein and TH protein compared with sham group but a slight increase compared with day 90. Representative images of the sympathetic nerve fascicles at different time points are shown in Fig. 6.

### Expression of the TH and S100 proteins in renal arteries, and norepinephrine concentration in the renal tissue

**Fig. 7:**
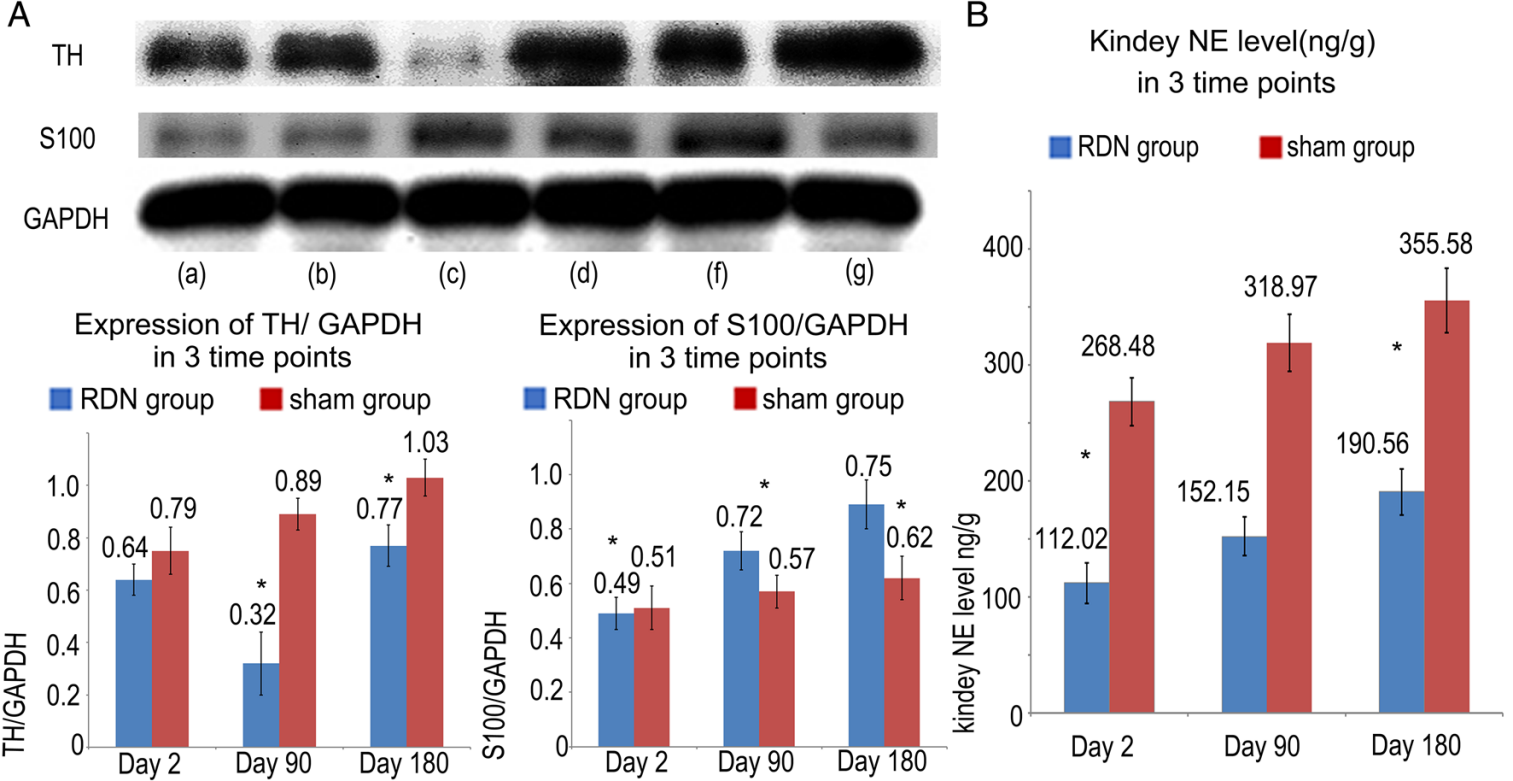
Changes in TH, S100 and NE levels at 4 time points. **A** Representative images of western blots for the TH and S100 proteins in renal arteries. **a**, **c**, **e**, **g** The RDN group at baseline and days 2, 90 and 180, respectively. **b**, **d**, **f**, **h**. The sham group at baseline and days 2, 90 and 180, respectively. **B** Changes in the renal NE level at the 3 time points. *P < 0.05 compared with the sham group

Two days after surgery, the expression of both the TH and S100 proteins was not significantly different between the RDN group and sham group. Significantly lower TH expression was observed in the RDN group than in the sham group at 90 days and 180 days after surgery, while the expression of the S100 protein in the atria was increased at day 90 and day 180 after RDN compared with the sham group. The NE concentration in renal tissues from RDN group was significantly lower than in the sham group (112.02 ± 17.34 ng/g vs 268.48 ± 20.61 ng/g, *P* < 0.001, 152.15 ± 16.61 ng/g vs 318.97 ± 24.84 ng/g, *P* < 0.001, and 190.56 ± 19.78 ng/g vs 355.58 ± 27.65 ng/g, *P* = 0.001, respectively) (Fig. 7).

## Discussion

According to several studies, RDN may be an efficient approach for treating resistant hypertension. Despite the negative results of SYMPILICTY HTN-3 [4], the newly discovered findings from the SPYRAL HTN-ON MED [5] and RADIANCE-HTN SOLO [6] studies have encouraged all researchers. The ablation devices used in these two studies may be one of the significant factors contributing to the success of RDN. In the present study, we performed laparoscopic-based perivascular RDN and a sham operation in two groups of minipigs. In the RDN group, the nerve fascicles contained in the renal arterial wall were successfully destroyed. Following high-fat diet intake, both SBP and DBP increase with the progression of obesity, consistent with several previous studies [7, 8]. However, SBP, ΔSBP, DBP, ΔDBP and NE levels in the renal tissue of the RDN group were significantly lower than in the sham group in the current study, indicating that laparoscopic-based perivascular RDN may be a feasible strategy to prevent the emergence and development of hypertension, and may be a potential treatment for hypertension. Our previous study [9] and other studies [10, 11] also confirmed that ablating renal nerves from the adventitia of the renal artery destroys the renal sympathetic nerve and suppresses the overactivated sympathetic nervous system.

NE, the main catecholamine neurotransmitter in the sympathetic nervous system, is an important indicator of the function of the sympathetic nerve fascicle. As shown in several studies [9, 12], RDN results in a marked decrease in the NE spillover rate, indicating that RDN disrupts the function of the sympathetic nerve fascicles in the renal arteries and suppresses the overactivated sympathetic nervous system. In the present study, significantly lower NE levels were observed in the renal tissue from the RDN group than in the sham group after the operation, indicating the decreased activity of sympathetic nervous system. This decrease was also confirmed by the low expression of TH detected using immunohistochemical staining and western blotting. TH is the enzyme responsible for catalyzing the conversion of the amino acid l-tyrosine to l-3,4-dihydroxyphenylalanine (L-DOPA), a precursor of dopamine, which, in turn, is a precursor for the important neurotransmitter NE. Lower expression levels of TH indicated a reduction in NE synthesis rates. During the self-healing process, TH expression was partially recovered at day 180 compared with day 2 and day 90, but was still lower than the sham group. This finding may indicate that perhaps the function of the injured peripheral nerve is partially but not completely restored, despite the regeneration of small nerve fascicles in the arterial wall.

HE staining indicated vacuolization and nuclear pyknosis of the affected nerve fascicles, and the endoneurium became disorganized after RDN, followed by endoneurial and perineural fibrosis, the formation of a thick layer and disordered nerve regrowth at the ablation site. Most of the affected nerve fascicles were enlarged visually, which may be caused by fibroblast proliferation and fibrous scar formation. Moreover, the regenerating nerve fascicles infiltrated into the surrounding tissue. This phenomenon is consistent with the process of peripheral nerve injury and regeneration [13] and has also been reported in other studies [14]. The S100 protein is a marker of SCs, which plays an important role in the development and regeneration of nerve fascicles after peripheral nerve injury. The increased expression of the S100 protein indicates the proliferation of SCs, as well as the repair and regeneration of renal nerve fascicles. Compared with the results obtained on day 2, the expression of the S100 protein in the RDN group was significantly increased on day 90 and day 180, which may be caused by the new growth of nerve fibers.

Undoubtedly, with the promotion of ablation devices, the availability of catheter-based endovascular RDN, which is performed in an interventional manner, may be substantially improved, similar to the results of SPYRAL HTN-ON MED and RADIANCE-HTN SOLO trials. Nevertheless, the problems associated with this procedure should not be ignored. Currently, although the number of patients who undergo percutaneous endovascular RDN is limited and the follow-up period is short, some side effects have been reported [15], i.e., lumen stenosis, atrial wall hematoma, intimal tears, intraluminal thrombosis, intima-media thickening [16–19], etc. Most of these factors may be caused by the direct delivery of RF energy or stimulation of the endothelium by the wire and catheter. Theoretically, by decreasing the direct stimulation with RF energy, wire and catheter of the arterial intima, perivascular RDN may be much safer than percutaneous endovascular RDN.

Based on the results of routine renal angiography, we performed OCT scanning, which has a high axial resolution of 10–20 μm [20], to visualize some vascular lesions that are not apparent on angiography [21] and to verify the safety of laparoscopic-based perivascular RDN. According to the results of renal arteriography, OCT and pathological evaluation, we did not observe any obvious injury or other serious complications in the 180-day study of the effects of laparoscopic-based perivascular renal sympathetic nerve denervation, although spasms were observed in the transient renal arteries either at the ablation sites or at other sites immediately after RDN in some segments. This phenomenon has also been reported in other studies of endovascular RDN [21], and while the mechanism is unknown, it may partially be due to the RF stimulation of the smooth muscle of the renal arterial wall. The spasms disappeared in the follow-up renal arteriography and OCT scans.

No statistically significant differences were observed in the serum creatinine, cystatin C and neutrophil gelatinase-associated lipocalin levels at each time point, indicating that laparoscopic-based perivascular RDN did not obviously alter renal function.

In summary, laparoscopic-based perivascular RDN destroys the nerve surrounding renal arteries and decreases the increased SBP, DBP and secretion of the sympathetic-active molecule NE in obese pigs, but does not exert obvious effects on the arterial lumen and arterial intima. The intima is crucial for maintaining a normal vascular structure and function and exerts atheroprotective effects in vivo by releasing substances that promote anticoagulation, inhibit inflammation, and induce vasodilatation [22], causing injury to the endothelium that may be associated with a high risk of renal artery atherosclerosis.

Without contrast agent infusion, X-ray exposure, or renal arterial intima direct stimulation, laparoscopic-based perivascular RDN also represents an alternative to traditional RDN, if needed by patients.

## Conclusions

As shown in the present study, laparoscopic-based perivascular RDN successfully destroys sympathetic nerve fascicles and prevents the increasing trends in SBP, DBP, renal NE levels and TH expression in the renal arteries of Bama minipigs fed a high-fat diet, indicating that laparoscopic-based perivascular RDN prevents the occurrence and development of hypertension and may be an alternative strategy for controlling blood pressure.

### Limitation

This study has some limitations. One limitation is that we did not establish a hypertension model before the operation, because of the long duration. We fed minipigs a high-fat diet after RDN to increase their weight and blood pressure, and our aim was to analyze the effect of laparoscopic-based perivascular RDN on the increase in BP.

This preclinical study performed was in a small number of Bama minipigs. The results also should be confirmed in additional high-quality trials with large samples and longer follow-up. Moreover, as an initial attempt to confirm the feasibility of this approach, specialized catheters or forceps for perivascular RDN are unavailable, and thus appropriative devices must be designed and produced before this method is applied in clinical practice.

## Data Availability

The datasets are available upon reasonable request.

## Abbreviations

BP: Blood pressure
NE: Norepinephrine
RDN: Renal sympathetic nerve denervation
RF: Radio-frequency
HE: Hematoxylin–eosin
